# Cultivar-Specific Defense Responses in Wild and Cultivated Squash Induced by Belowground and Aboveground Herbivory

**DOI:** 10.1007/s10886-024-01523-9

**Published:** 2024-06-24

**Authors:** Wenfeng Ye, Leandro Di Caprio, Pamela Bruno, Charlyne Jaccard, Carlos Bustos-Segura, Carla C. M. Arce, Betty Benrey

**Affiliations:** 1 Laboratory of Fundamental and Applied Research in Chemical Ecology, Institute of Biology, University of Neuchâtel, Neuchâtel, Switzerland; 2 CAS Key Laboratory of Insect Developmental and Evolutionary Biology, CAS Center for Excellence in Molecular Plant Sciences, Shanghai Institute of Plant Physiology and Ecology, Chinese Academy of Sciences, Shanghai, China; 3 Laboratory of Evolutionary Entomology, Institute of Biology, University of Neuchâtel, Neuchâtel, Switzerland; 4 Plant Production Systems, Route Des Eterpys 18, 1964 Agroscope, Conthey Switzerland; 5 Sensory Ecology, Institute of Ecology and Environmental Sciences of Paris, INRAE, Versailles, France

**Keywords:** Cucurbitacin, Trichomes, Plant domestication, Plant defense, *Diabrotica balteata*, *Cucurbita argyrosperma*

## Abstract

Plant domestication often alters plant traits, including chemical and physical defenses against herbivores. In squash, domestication leads to reduced levels of cucurbitacins and leaf trichomes, influencing interactions with insects. However, the impact of domestication on inducible defenses in squash remains poorly understood. Here, we investigated the chemical and physical defensive traits of wild and domesticated squash (*Cucurbita argyrosperma*), and compared their responses to belowground and aboveground infestation by the root-feeding larvae and the leaf-chewing adults of the banded cucumber beetle *Diabrotica balteata* (Coleoptera: Chrysomelidae). Wild populations contained cucurbitacins in roots and cotyledons but not in leaves, whereas domesticated varieties lacked cucurbitacins in all tissues. Belowground infestation by *D. balteata* larvae did not increase cucurbitacin levels in the roots but triggered the expression of cucurbitacin biosynthetic genes, irrespective of domestication status, although the response varied among different varieties. Conversely, whereas wild squash had more leaf trichomes than domesticated varieties, the induction of leaf trichomes in response to herbivory was greater in domesticated plants. Leaf herbivory varied among varieties but there was a trend of higher leaf damage on wild squash than domesticated varieties. Overall, squash plants responded to both belowground and aboveground herbivory by activating chemical defense-associated gene expression in roots and upregulating their physical defense in leaves, respectively. While domestication suppressed both chemical and physical defenses, our findings suggest that it may enhance inducible defense mechanisms by increasing trichome induction in response to herbivory.

**Supplementary Information:**

The online version contains supplementary material available at 10.1007/s10886-024-01523-9.

## Introduction

Plant domestication is an evolutionary process wherein wild plants are artificially selected to alter traits beneficial for human use (Diamond 2002; Purugganan and Fuller 2009). This process is regarded as one of the most important developments in human history, enabling the shift from nomadic lifestyles to sedentary societies and incited the rise of modern civilization (Diamond 2002; Ross-Ibarra et al. 2007; Larson et al. 2014). Domestication often leads to a genetic bottleneck, resulting in domesticated plants having less genetic diversity than their wild counterparts (Meyer and Purugganan 2013; Gaut et al. 2015). This reduced genetic diversity coupled with trade-offs between growth and defense traits, can compromise crop resistance against pests (Wright et al. 2005; Yamasaki et al. 2005; Flint-Garcia 2013). In nature, plants employ various defense mechanisms against herbivores, including physical barriers and defensive chemicals to directly deter them (Kariyat et al. 2017; Erb and Reymond 2019). Additionally, plants can indirectly defend themselves by emitting herbivore-induced volatiles that attract natural enemies of insect pests (Turlings and Erb 2018) or by secreting extrafloral nectar that recruit protective bodyguards (Heil 2015). However, domestication has altered plant defense traits by influencing the expression of individual defensive genes or through selection on quantitative traits (Chen et al. 2015). Generally, domestication consistently reduces plant resistance traits and impacts plant–insect interactions (Chen et al. 2015; Whitehead et al. 2017; Fernandez et al. 2021). Domestication may also enhance the defenses of crops against specific herbivores (Yahiaoui et al. 2006; Gaillard et al. 2018). Importantly, the effect of domestication on plant defenses varies depending on species, genotype-, tissue- and pest specificity (Turcotte et al. 2014; Whitehead et al. 2017; Gaillard et al. 2018; Shlichta et al. 2018; Jaccard et al. 2021).

The genus *Cucurbita* L. (squash, pumpkins, and some gourds; Cucurbitales: Cucurbitaceae), native to the Andes and Mesoamerica, is one of the earliest domesticated plant groups (Smith 1997; Piperno and Stothert 2003). Domestication in *Cucurbita* has consisted of several independent events, resulting in five main domesticated species (Nee 1990; Sanjur et al. 2002; Castellanos-Morales et al. 2018). Each *Cucurbita* crop followed a distinct selection path to cater to local preferences and specific needs, certain aspects of the domestication syndrome, such as the reduction of plant resistance, are shared. These include the loss of defensive metabolites like cucurbitacins and the decrease in physical and chemical barriers like trichomes (Chomicki et al. 2020; Barrera-Redondo et al. 2021).

Cucurbitacins are a group of triterpenoid compounds predominant in the Cucurbitaceae family. These bitter secondary metabolites are toxic and antifeedant to many herbivores (Da Costa and Jones 1971; Ferguson and Metcalf 1985; Tallamy et al. 1997). However, cucurbitacins can also serve as attractants for several well-adapted phytophagous beetles such as Chrysomelidae (Chambliss and Jones 1966; Lewis and Metcalf 1996; Jaccard et al. 2021, 2023). Cucurbitacin biosynthetic genes and the cucurbitacin biosynthesis pathway have been identified in several cucurbit plants. In cucumber (*Cucumis sativus*), melon (*Cucumis melo*) and watermelon (*Citrullus lanatus*), tissue-specific transcription factors activate the initiation of cucurbitacin biosynthesis in fruits, leaves and roots, respectively. The loss of fruit bitterness is caused by the downregulation of fruit-specific transcription factors (*Bt*, *bitter fruit*) via mutation in their promoter regions (Shang et al. 2014; Zhou et al. 2016). In *Cucurbita pepo*, a single locus *Bi-4* has been found to play an essential role in the cotyledon cucurbitacin accumulation in seedling development through transport and regulation of cucurbitacins (Brzozowski et al. 2020). In a recent study, we found that domestication of *Cucurbita argyrosperma* strongly decreased the contents of cucurbitacins in roots and cotyledons by downregulating the expression of cucurbitacin biosynthetic genes (Jaccard et al. 2023). While extensive research has focused on the biosynthesis and regulation of cucurbitacin (Shang et al. 2014; Zhou et al. 2016; Brzozowski et al. 2020), comparatively little is known about its induction in response to herbivory and how domestication affects this process. Cucurbitacin has been identified as an inducible defensive compound in previous studies (Tallamy 1985; Agrawal et al. 1999). However, subsequent research has revealed that the induction of cucurbitacin biosynthesis may be contingent upon factors such as plant species, tissue type, and the specific herbivore involved. For example, in cucumber, herbivory by *Acalymma vittatum* or *Diabrotica balteata* larvae fails to increase cucurbitacin concentration in roots and leaves (Milano et al. 2015; Bruno et al. 2023), whereas spider mite feeding induces increased levels of cucurbitacin in locally damaged cotyledons and systemically in undamaged first true leaves (Agrawal et al. 1999). Furthermore, feeding by the striped cucumber beetle *A. vittatum* does not trigger cucurbitacin production in leaves and cotyledons of *Cucurbita pepo* (Brzozowski et al. 2020).

The trichome density of *Cucurbita* plants is another defensive feature that is commonly reduced during domestication (Barrera-Redondo et al. 2021). Trichomes, hair-like epidermal protuberances found on leaves, stems, and various plant organs, play a defensive role in many plant species (Wagner 1991; Kang et al. 2010; Andama et al. 2020; Kaur and Kariyat 2023). Trichomes are morphologically classified as either non-glandular or glandular (Wang et al. 2021). Non-glandular trichomes defend against herbivores by hindering their movement and deterring feeding (Riddick and Wu 2011; Kariyat et al. 2017; Kaur and Kariyat 2023). Glandular trichomes on the other hand, synthesize and secrete defensive compounds to protect against herbivores (Weinhold and Baldwin 2011; Bleeker et al. 2012; Sasse et al. 2016). Moreover, many plant species increase trichome formation in response to herbivore attack, mechanical damage, or phytohormone treatment (Agrawal 1999; Brian Traw and Dawson 2002; Dalin and Björkman 2003; Boughton et al. 2005; Zhang et al. 2019). In cucurbit plants, the profile and density of trichomes vary depending on the species and the purpose of domestication. In bottle gourd (*Lagenaria siceraria*) and cucumber (*Cucumis sativus*), non-glandular trichome density is greater than glandular trichome density (Kaur and Kariyat 2023). In squash, simple and non-glandular trichomes were identified on the leaves of varieties domesticated for consumption or ornamental purposes (Jaccard et al. 2021). However, some leaf herbivores exhibit similar performance, measured by survival and relative growth rate, on *Cucurbita* plants with different trichome densities (Jaccard et al. 2021; Burgueño et al. 2023).

In this study, we examined the impact of domestication on the chemical and physical resistance traits of *Cucurbita argyrosperma* plants and further explored how domestication influences the inducible plant defenses. We proposed two competing hypotheses: (1) Considering the evolutionary history of wild plants coping with herbivore pressure, it is possible that wild plants may exhibit higher levels of inducible defenses compared to their domesticated relatives. (2) Conversely, given that wild plants commonly possess robust constitutive defensive traits, the enhancement of these defenses through induction may only be evident in domesticated plants compared to their wild counterparts. To test these hypotheses, we compared the induction of plant defense responses in wild and domesticated plants in response to both belowground and aboveground herbivory. Specifically, we analyzed cucurbitacin levels in various plant tissues to investigate the impact of belowground herbivory by *Diabrotica balteata* larvae on cucurbitacin biosynthesis in the roots of different squash varieties. Additionally, we assessed the leaf trichome density of wild and domesticated squash plants to determine whether aboveground herbivory by *D. balteata* adults induced leaf trichome formation in different squash varieties. This study offers valuable insights into the role of domestication in altering both constitutive and inducible defensive traits in plants.

## Materials and Methods

### Plants

*Cucurbita argyrosperma* Huber, commonly known as cushaw pumpkin, pipiana squash, or silver-seed gourd is a species native to Mexico of cultural and economic local importance (Sanjur et al. 2002; Kates et al. 2017; Barrera-Redondo et al. 2021). Three wild *C. argyrosperma* populations and three domesticated ornamental varieties were used for the experiments (Fig. S1). Seeds of the wild populations (Wild Altitude [Walt]: 16°05′48.9′′ N, 97°05′02.4′′ W; Wild Bird lagoon [Wbi]: 15°49′14.9′′ N, 97°01′52.4′′ W; Wild Los Lanches [Wll]: 15°55′22.3′′ N, 97°08′19.3′′ W) were collected along the coast of Puerto Escondido, Oaxaca state, Mexico. While seeds of the three domesticated varieties (Ornamental Cushaw Orange Striped [OCS], Ornamental Cushaw Tricolor [OCT], and Ornamental Cushaw White [OCW]) were obtained from KCB-Samen GmbH, Basel, Switzerland. Seeds of domesticated varieties were germinated individually in plastic pots (diameter 12 cm; volume 630 mL) filled with a mixture of commercial potting soil (Einheitserde Classic, PATZER ERDEN GmbH, Sinntal-Altengronau, Germany) and sand (Sable Capito, diameter 1–4 mm, Landi, Dotzigen, Switzerland) (soil: sand 70%:30%; v/v). To enhance germination of the wild seeds, we delicately pierced and scratched the seed coat using a punch and a nail file and then placed them in a Petri dish with wet cotton for one week in an incubator at 28 °C in the dark. Upon germination, wild seeds were individually transferred to pots and grown following the same procedure as seeds from domesticated varieties. All plants were grown under controlled conditions (24 ± 5 °C; 60% relative humidity; 16-/8-h light/dark photoperiod) in the greenhouse and watered every other day. As wild seeds take longer to germinate, they were incubated for germination 10 days before the seeds from domesticated varieties to control for differences in growth. Fifteen-day-old plants with two fully developed leaves were used for all experiments.

### Insect

The banded cucumber beetle, *Diabrotica balteata* LeConte (Coleoptera: Chrysomelidae), originated in the tropical Americas, is an important agricultural pest species of a variety of crops, including cucurbits (Saba 1970). The larvae of *D. balteata* mainly feed on the roots and tubers of plants, while adult beetles eat most plant parts, including leaves, fruits, flowers, and seedling cotyledons (Pitre and Kantack 1962). Eggs of *D. balteata* were provided by Syngenta (Stein, Switzerland) and kept in Petri dishes until hatching. Larvae of *D. balteata* were maintained in a quarantine facility at the University of Neuchâtel on freshly germinated maize roots (hybrid DFI 45321, DSP, Delley, Switzerland) under controlled conditions (25 ± 2 °C; 60% ± 5% relative humidity; 16-/8-h light/dark photoperiod). Second-instar larvae were used in the root herbivory experiment to evaluate the effect of root herbivory on cucurbitacin accumulation in squash roots. Adults were kept in cages with moistened cotton and fed on pollen (Hoyer GmbH, Polling, Germany) before experiments. Seven-day-old adults were used in the leaf herbivory experiment to evaluate the effect of herbivory on the systemic induction of trichomes.

### Plant Treatments

To assess the effect of root and leaf herbivory on plant defense in both wild and domesticated squash, we conducted two separate induction experiments (Fig. S2). Squash plants were subjected to infestation either by root-feeding *D. balteata* larvae or leaf-feeding *D. balteata* adults. The root responses were assessed by measuring cucurbitacin contents and the transcription levels of cucurbitacin biosynthesis-related genes. Leaf responses were evaluated by measuring leaf trichome density.

1. **Root herbivory experiment**
  Fifteen-day-old squash plants were transplanted into individual plastic bags, and ten second-instar larvae were released onto the soil surface around the stem of each plant to infest the roots. To prevent larval escape, the plastic bag was then sealed at the stem base using parafilm. Five plants of each population and variety were infested with larvae. Three uninfested plants of each population and variety were used as controls. After 24 h infestation, the larvae were removed from the roots, and the entire root system was harvested, flash-frozen in liquid nitrogen, and stored at -80 °C until further analysis.
2. **Leaf herbivory experiment**
  Fifteen-day-old squash plants with two expanded leaves were used in this experiment. To prevent herbivory by *D. balteata* adults on the stem, aluminum foil was placed beneath the cotyledon. Plants were kept in individual cages (30 × 30 × 30 cm; Vermandel products, Hulst, The Netherlands) under controlled conditions (25 ± 2 °C; 60% ± 5% relative humidity; 16-/8-h light/dark photoperiod). The buds with emerging leaves were protected with woven nylon mesh to avoid *D. balteata* adult herbivory. Two adults of *D. balteata* (one week after emergence from the pupae) were introduced into each cage and allowed to feed on leaves and cotyledons for four hours daily. After four days, photographs of the two exposed leaves were taken, and ImageJ was used to assess leaf consumption by *D. balteata* adults. Ten days following the four-day herbivory period, the trichome density of the three new leaves of each plant was determined. Five plants of each population and variety were infested with *D. balteata* adults. Three control plants of each population and variety were remained uninfested.

### Cucurbitacin Quantification

To examine the tissue-specific distribution of cucurbitacins in uninfested wild and domesticated *C. argyrosperma* plants, roots, cotyledons, and leaves were collected from three fifteen-day-old plants of three wild populations and three domesticated squash varieties. To assess the effect of root herbivory on cucurbitacin levels in various squash plants, the entire root systems were harvested from control plants that remained uninfested or from plants damaged by ten second-instar *D. balteata* larvae, which freely fed on them for 24 h.

Plant cucurbitacins were extracted and quantified following the method outlined by Jaccard et al. (2021, 2023). Tissue samples were flash-frozen in liquid nitrogen and then ground into a fine powder. A 100 mg portion of the powder was extracted with 1 ml of methanol (99.99%). Samples were lysed in a TissueLyser (Qiagen, Hilden, Germany) for 4 min at 30 Hz with 5 glass beads (1.25–1.65 mm diameter). Lysed samples were centrifuged at 20,913 × g (14,000 rpm) for 5 min and 700 µL of supernatants were diluted with 300 µL of Milli-Q water. Quantification of cucurbitacins was performed using an ultra-high performance liquid chromatography-quadrupole time-of-flight mass spectrometry (UHPLC-QTOFMS) system with an Acquity UPLC™ coupled to a Synapt G2 QTOF mass spectrometer (Waters, Milford, MA, USA). Peaks of known cucurbitacins were automatically integrated using Quanlynx™ with a 0.1 min chromatographic window centered on each component’s retention time and a 0.02 Da mass window centered on the (M + HCOO) ion. Quantification of all cucurbitacins was done by external calibration using cucurbitacin B as a standard. The cucurbitacin concentration is expressed in micrograms (μg) per gram (g) of plant material.

### RNA Extraction and Quantitative Real-Time PCR Analysis

Freshly harvested roots were ground in liquid nitrogen, and RNA was isolated using the GeneJET Plant RNA Purification Mini Kit (Thermo Fisher Scientific Baltics UAB, Vilnius, Lithuania) following the manufacturer’s protocol. Complete DNA removal was achieved using the RNase-Free DNase Set (QIAGEN, Hilden, Germany). Subsequently, each total RNA sample (500 ng) was reverse-transcript using the GoScript™ Reverse Transcription System (Promega).

We used amino acid sequences of cucurbitacin biosynthetic genes from cucumber, melon, and watermelon (Zhou et al. 2016) as queries to search orthologs in *C. argyrosperma* genome database (http://cucurbitgenomics.org/v2/organism/27) (Barrera-Redondo et al. 2019) by using BLASTp. This search revealed nine putative cucurbitacin biosynthetic genes, including one candidate for cucurbitadienol synthase gene (*Carg11552*), one for acetyltransferase gene (*Carg03796*), and seven for cytochrome P-450 enzymes genes (*Carg03795*, *Carg03797*, *Carg06672*, *Carg07313*, *Carg08824*, *Carg11550*, *Carg11551*) (Supplemental Table S1) were found in *C. argyrosperma* genome. From these, we selected six putative cucurbitacin biosynthetic genes and assessed their relative expression levels in the roots of uninfested and *D. balteata* larvae-infested plants of both wild and domesticated squash. Primers used for real-time qPCR were designed by using Primer-BLAST (https://www.ncbi.nlm.nih.gov/tools/primer-blast/) and listed in Supplemental Table S2.

Real-time qPCR was performed on the Rotor-Gene™ 6000 (Corbett Research) platform. The qPCR mix consisted of 10 μl GoTaq® qPCR Master Mix (Promega), 8.2 μl H_2_O, 0.4 μl each primer (10 μM), and 1 μl of cDNA sample. The qPCR was performed using 50 cycles with the following temperature curve: 10 s at 95 °C, 20 s at 65 °C and 2 s 72 °C. The melt curve was obtained by ramping from 72 °C to 99 °C, rising by 1 °C each step, and waiting for 5 s for each step afterward. The actin gene (GenBank accession number: HM594170) was used as the reference. For each variety, five independent biological replicates of herbivore-damaged plants and three replicates of undamaged control plants were analyzed, after which the average threshold cycle (Ct) per sample was calculated. For the expression analysis of each gene, samples from undamaged OCT variety were designated as calibrators. The relative expression levels were determined using the 2^−△△Ct^ method (Livak and Schmittgen 2001).

### Trichome Density Quantification

To assess trichome density in various wild and domesticated *C. argyrosperma* plants, the number of trichomes in an area of 9 mm^2^ on the adaxial side of the second leaf at the central part of the leaf base was recorded using a microscope (Nikon SMZ 1000) coupled with microscope camera control (Nikon Digital Sight Ds-l1). We only counted trichomes in the adaxial side of the leaf, as trichome numbers are correlated between the adaxial and abaxial side (Jaccard et al. 2021). Captured images were analyzed with ImageJ software (ImageJ 1.53j 13 May 2021 version, US). Fifteen individual squash plants per wild population and domesticated variety were used to calculate trichome density.

To evaluate the systemic induction of trichomes in response to leaf herbivory by *D. balteata* adults in different squash plants, the trichome density of the three newly emerged leaves from undamaged control plants (n = 3) or plants damaged by two seven-day-old adults (n = 5) per variety and wild population was determined using the same procedure as described above.

### Statistical Analysis

Data analyses were carried out with R statistical software (v. 4.3.0; R Development Core Team 2019). To compare the levels of constitutive cucurbitacin levels among six different varieties, generalized linear model (GLM) with Gamma distribution and log link function followed by Tukey post-hoc test for multiple comparisons were used. Additionally, we used generalized linear mixed model (GLMM) with Gamma distribution and log link function followed by Tukey post-hoc test to compare the cucurbitacin contents between wild and domesticated squash, with varieties set as random factor. The induction of cucurbitacin was analyzed using GLM with Gamma distribution and log link function followed by Tukey post-hoc test for multiple comparisons, with infestation treatment and varieties as explanatory variables.

Differences in gene expression between uninfested and infested squash roots in each variety were compared using Student’s *t*-test. For the comparison of herbivory-induced gene expression data pooled by domestication status, generalized linear mixed models (GLMM) with Gamma distribution and log link function were applied, followed by Tukey post-hoc test, with infestation treatment and domestication status as explanatory variables, and varieties as random factor.

To compare the trichome density and consumed leaf area among plant varieties, generalized linear mixed models (GLM) with Gamma distribution and log link function followed by Tukey post-hoc test were used. Similarly, to compare the trichome density between domestication status and the consumed leaf area, GLMM with Gamma distribution and log link function followed by Tukey post-hoc test were used, with domestication status as explanatory variable and varieties as random factor.

The herbivory-induced trichome formation was analyzed with a GLMM followed by Tukey post-hoc test for multiple comparisons, with infestation treatment and domestication status as explanatory variables, and varieties as random factor. Differences in trichome density on each newly emerged leaf within each plant variety between uninfested and infested squash were compared using Student’s *t*-test.

## Results

### Domestication Decreases Cucurbitacin Content in Roots and Cotyledons of Squash

**Fig. 1:**
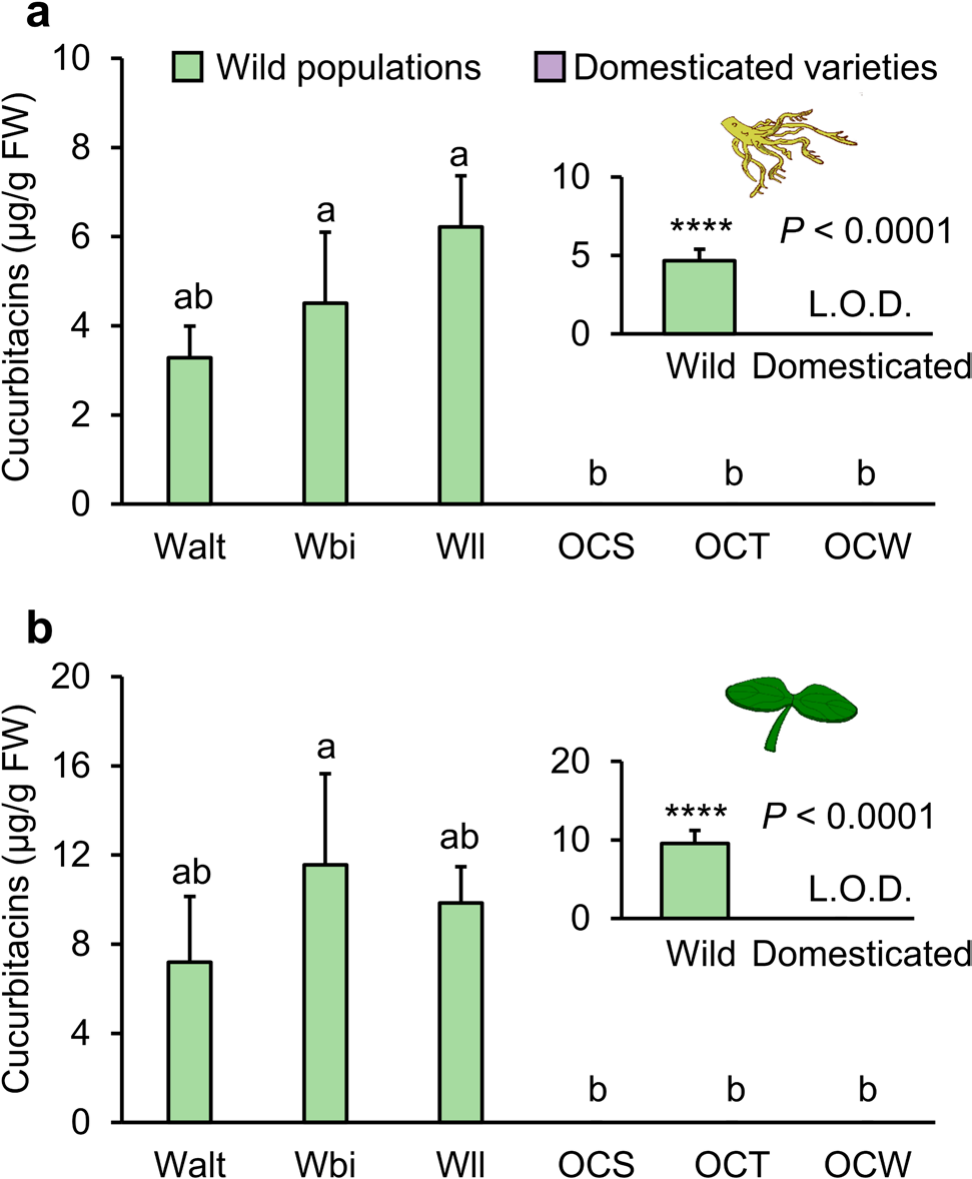
Cucurbitacin levels in different tissues of wild populations of *Cucurbita argyrosperma* and domesticated ornamental varieties. Concentrations of cucurbitacin in roots (**a**) and cotyledons (**b**) of three wild populations (Walt, Wbi, and Wll, in green) and three ornamental varieties (OCS, OCT, and OCW in purple, no detection) are shown in micrograms / gram FW: fresh weight. Error bars represent standard error of the mean (*n* = 3). Different letters indicate significant differences among wild populations and domesticated varieties (*P* < 0.05). *P* values are given for comparison among varieties with generalized linear model (GLM) with a Gamma distribution and log link function followed by Tukey post-hoc test. Asterisks indicate significant differences between wild and domesticated varieties (**** *P* < 0.0001). *P* values are given for comparison between domestication status with mixed generalized linear model (GLMM) with Gamma distribution and log link function followed by Tukey post-hoc test. Cucurbitacin levels in roots and cotyledons of domesticated plants are lower than the limit of detection (L.O.D.)

In total, five main cucurbitacins were found in squash roots and cotyledons (Table S3). Cucurbitacin level in the roots were significantly different among the six plant types (Chisq = 50.885, df = 5, *P* = 9.129e-10; Fig. 1a). When we compared wild and domesticated plants, domestication status explained the difference in cucurbitacin content (Chisq = 2700.8, df = 1, *P* < 2.2e-16; Fig. 1a). No significant differences in cucurbitacin content in roots among the three wild populations (Walt—Wbi, *P* = 0.9055; Walt—Wll, *P* = 0.2211; Wbi—Wll, *P* = 0.7179; Fig. 1a), whereas a complete lack of cucurbitacins was found in all three domesticated ornamental varieties (OCS—OCT, *P* = 1; OCS—OCW, *P* = 1; OCT—OCW, *P* = 1; Fig. 1a). Similarly, cucurbitacin levels in the cotyledons were significantly different among the six plant types (Chisq = 31.369, df = 5, *P* = 7.921e-06; Fig. 1b). The three wild populations contained similar levels of cucurbitacin in the cotyledons (Walt—Wbi, *P* = 0.7100; Walt—Wll, *P* = 0.9467; Wbi—Wll, *P* = 0.9920; Fig. 1b), whereas no cucurbitacins were found in the cotyledons of the domesticated varieties (OCS—OCT, *P* = 1; OCS—OCW, *P* = 1; OCT—OCW, *P* = 1; Fig. 1b). When data was pooled by domestication status, cucurbitacin content in cotyledons was higher in the wild populations than in the domesticated varieties (Chisq = 5221.4, df = 1, *P* < 2.2e-16; Fig. 1b). No cucurbitacins were detected in the leaves of both wild and domesticated squash plants.

### Belowground Infestation by *D. balteata* Larvae Does Not Increase Cucurbitacin Content in Roots of Squash

**Fig. 2:**
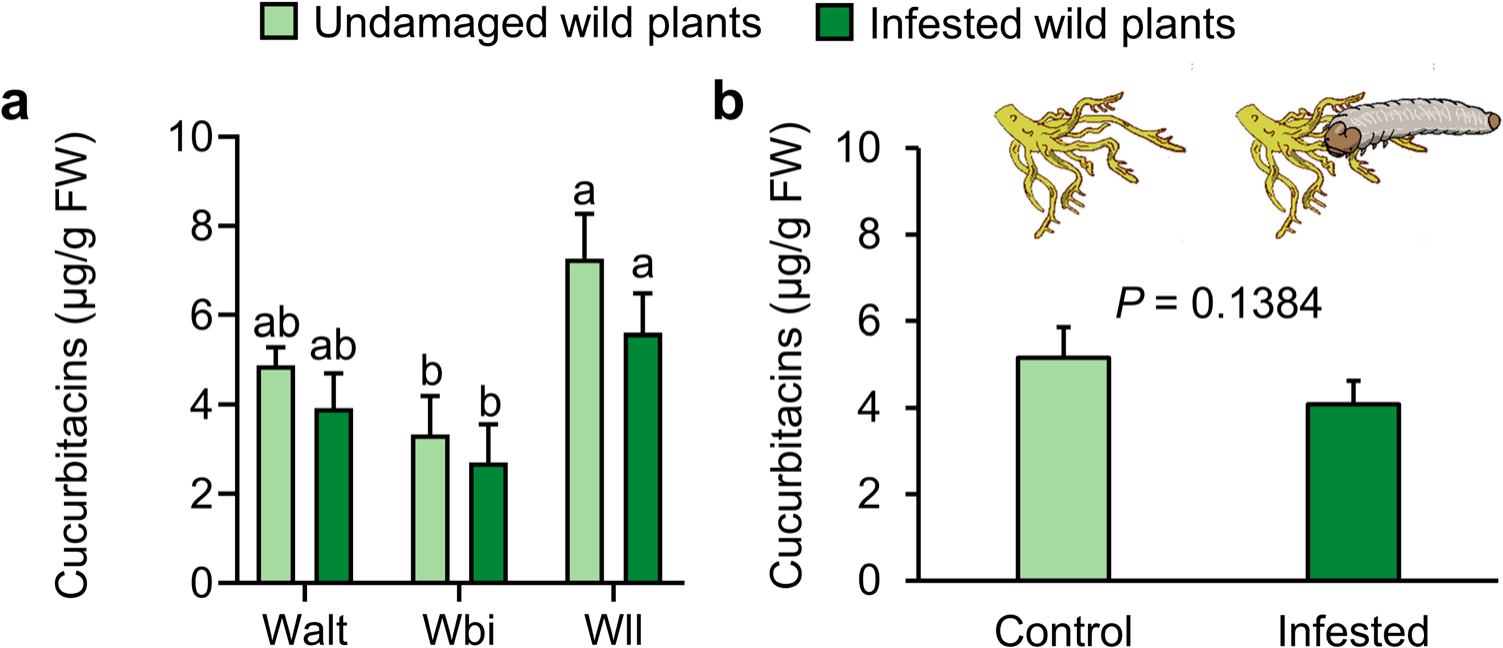
Belowground herbivory by *Diabrotica balteata* larvae does not increase the level of cucurbitacin in roots of *Cucurbita argyrosperma* plants. Root samples were collected from squash plants that were not infested (controls) or after 24 h of belowground infestation by 10 second-instar *D. balteata* larvae. Concentrations of cucurbitacin in roots of three wild populations (Walt, Wbi, and Wll) are shown in micrograms / gram FW: fresh weight (**a**). Error bars represent standard error of the mean (for controls, *n* = 3; for root infestation, *n* = 5). Different letters indicate significant differences among treatments (*P* < 0.05). Data were pooled based on infestation status and shown in (**b**). *P* values are given for comparison among varieties with a generalized linear model (GLM) with Gamma distribution and log link function followed by Tukey post-hoc test

To investigate whether belowground herbivory induces the accumulation of cucurbitacin in squash roots, we compared the level of cucurbitacin in roots of uninfested plants to those infested by the root larvae of *D. balteata*. Root infestation by *D. balteata* larvae for 24 h did not lead to an increase in the level of cucurbitacin in roots of wild populations (Chisq = 2.1954, df = 1, *P* = 0.1384; Fig. 2). Additionally, no cucurbitacin was detected in roots of both uninfested and infested domesticated squash.

### Belowground Infestation by *D. balteata* Larvae Increases the Transcription of Cucurbitacin Biosynthesis Genes

To investigate whether the belowground infestation by *D. balteata* larvae activates cucurbitacin biosynthesis and to assess the impact of domestication in this process, we examined the relative expression levels of six candidate cucurbitacin biosynthetic genes in roots of uninfested and *D. balteata* larvae-infested plants, for both wild and domesticated squash. In wild squash plants, *D. balteata* larvae infestation increased the transcription level of *Carg11552*, *Carg06672*, and *Carg07313* in the wild population Wbi. Additionally, the expression of *Carg07313* was upregulated, while the transcription of *Carg03795* was downregulated in the wild population Wll following root damage. Conversely, in the wild population Walt, none of the tested genes were induced after belowground herbivory. Among the six tested genes, only *Carg07313* was significantly induced upon root herbivory in all three domesticated varieties (Fig. S3).

**Fig. 3:**
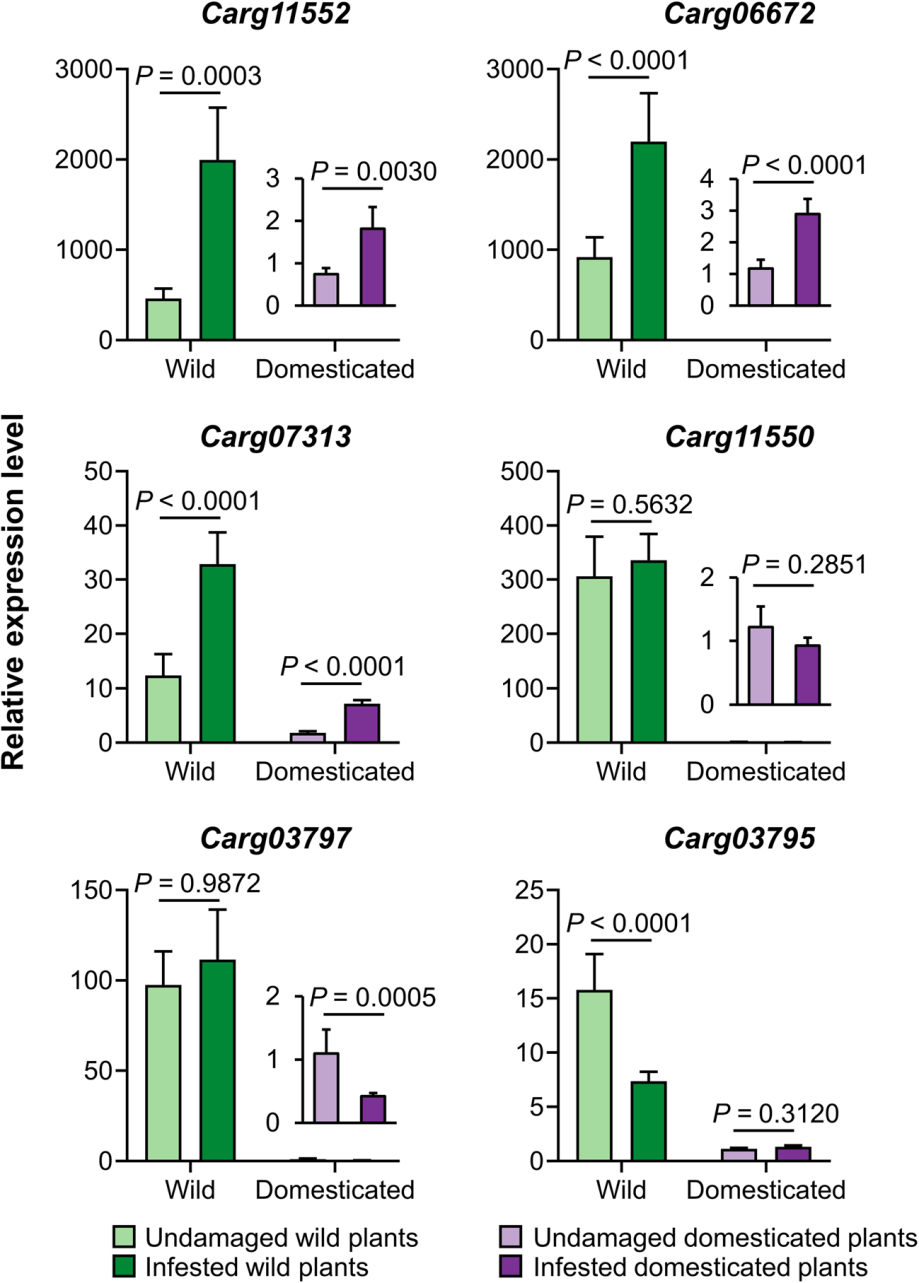
The effect of belowground herbivory by *Diabrotica balteata* larvae on the expression of cucurbitacin biosynthesis genes in roots of wild and domesticated squash plants. Mean transcript levels (+ SE) of six cucurbitacin biosynthesis genes in roots of wild population (Walt, Wbi, and Wll) and domesticated varieties (OCS, OCT, and OCW) of squash plants that were not infested or infested with 10 second-instar *D. balteata* larvae for 24 h. Error bars represent standard error of the mean (undamaged wild plants, *n* = 3 population × 3 replicates; infested wild plants, *n* = 3 population × 5 replicates; undamaged domesticated plants, *n* = 3 varieties × 3 replicates; infested domesticated plants, *n* = 3 varieties × 5 replicates). *P* values are given for comparison between domestication status with mixed generalized linear model (GLMM) with Gamma distribution and log link function followed by Tukey post-hoc test

While the expression level of cucurbitacin biosynthetic genes was notably lower in domesticated plants compared to wild squash, pooling the expression data as wild or domesticated revealed similar induced expression patterns in biosynthesis genes (*Carg11552*, *Carg06672*, and *Carg07313*) in response to root damage across plants with different domestication status (Fig. 3).

### Wild Squash has Higher Leaf Trichome Density Compared to Domesticated Plants, but Leaf Damage Caused by *D. balteata* Adults is not Reduced

**Fig. 4:**
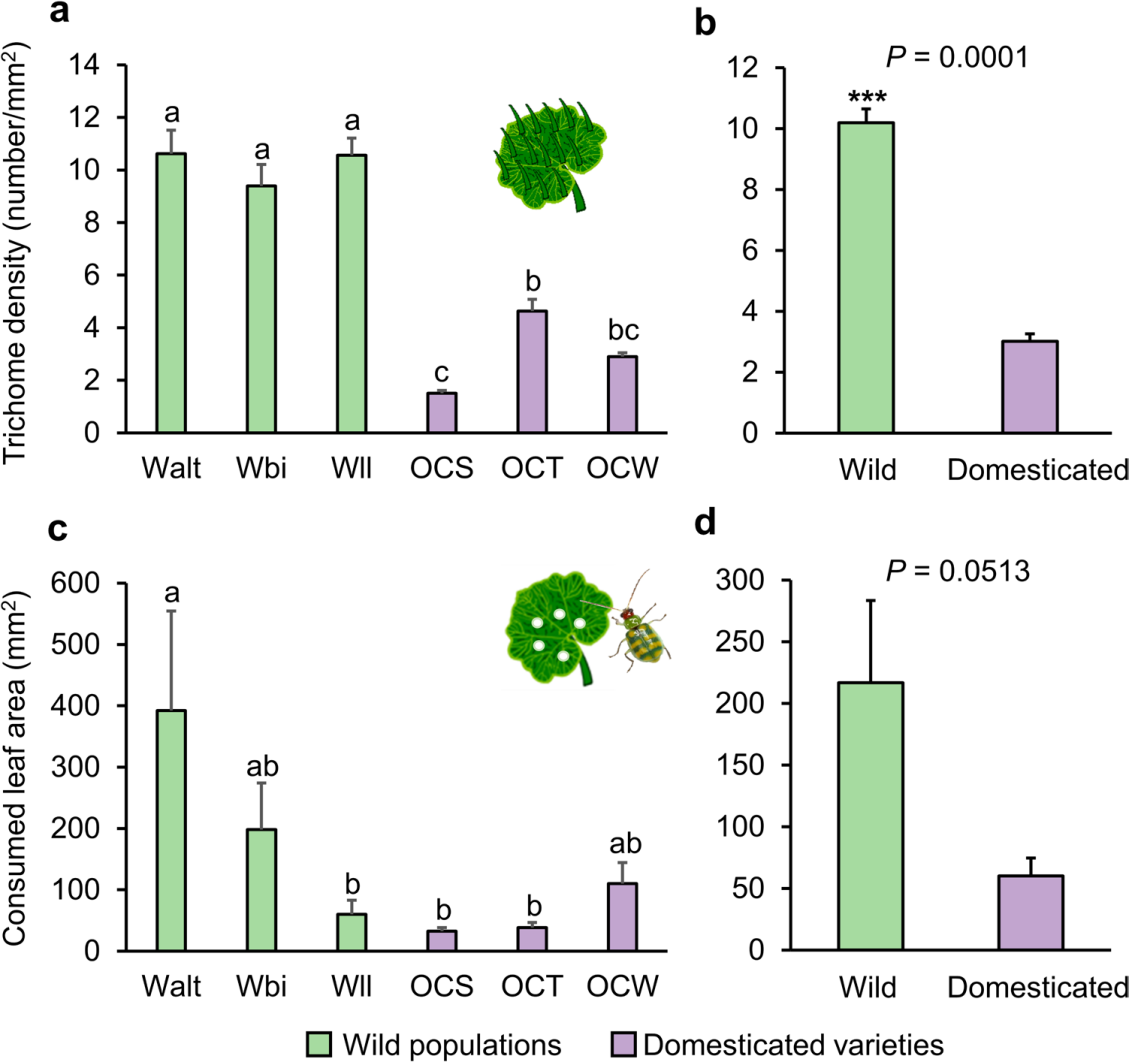
Wild populations of *Cucurbita argyrosperma* plants exhibit higher leaf trichome density but do not suffer less leaf damage than domesticated varieties when they were infested by *Diabrotica balteata* adults. (**a**) Trichome density (*n* = 15, ± SE) on the adaxial side of the second leaf of wild populations (Walt, Wbi, and Wll) and domesticated varieties (OCS, OCT, and OCW) of squash plants was presented by number of trichomes per square millimeter leaf area. Data were pooled based on domestication status and shown in (**b**). (**c**) Leaf consumption (*n* = 5, ± SE) by two *D. balteata* adults for four days (four hours per day) on wild and domesticated squash plants. Data were pooled based on domestication status and shown in (**d**). Different letters indicate significant differences among wild populations and domesticated varieties (*P* < 0.05).* P* values are given for comparison among varieties with generalized linear model (GLM) with Gamma distribution and log link function followed by Tukey post-hoc test. Asterisks indicate significant differences between wild and domesticated varieties (*** *P* < 0.001). *P* values are given for comparison between domestication status with mixed generalized linear model (GLMM) with Gamma distribution and log link function followed by Tukey post-hoc test

Simple and non-glandular trichomes were observed on the leaves of all wild populations and domesticated varieties. Overall, leaf trichome density was significantly higher in wild populations than in domesticated varieties (Chisq = 15.113, df = 1, *P* = 0.0001; Fig. 4a-b). The three populations of wild squash had similar trichome density (Walt—Wbi, *P* = 0.6957; Walt—Wll, P = 1; Wbi—Wll, *P* = 0.7330; Fig. 4a), whereas trichome density varied among domesticated varieties (OCS—OCT, *P* = 0.0047; OCS—OCW, *P* = 0.5659; OCT—OCW, *P* = 0.3145; Fig. 4a). Leaves of variety OCT presented the highest trichome density while variety OCS presented the lowest (Fig. 4a).

The consumption of leaf area by *D. balteata* adults varied among different wild and domesticated squash, and this variability could be partially explained by domestication status. Leaf consumption by *D. balteata* adults was highest for the wild population Walt and no significant difference was found among all the other wild and domesticated varieties (Fig. 4c). However, when data was pooled by domestication status, there was a trend of higher leaf damage on wild plants than domesticated varieties (Chisq = 3.7992, df = 1, *P* = 0.0513; Fig. 4d).

### Aboveground Herbivory by *D. balteata* Adults Induces an Increase in Leaf Trichome Density in Certain Squash Plants

To determine whether feeding by *D. balteata* beetles induces trichome formation on squash leaves, we compared the trichome density of the newly emerged leaves on infested plants with uninfested control plants, for both wild and domesticated squash. In wild populations, foliar feeding by adult *D. balteata* beetles did not result in an increased trichome density on leaves of each population, except for the 3rd leaf of Walt (*P* = 0.0179) (Fig. S4a-c). Among domesticated varieties, leaf feeding by *D. balteata* increased trichome density on the 4th leaf of OCS (*P* = 0.0483) as well as the 4th and 5th leaves of OCW (4th leaf: *P* = 0.0277; 5th leaf: *P* = 0.0108), while none of the leaves of OCT plants exhibited higher trichome density after herbivory (Fig. S4d-f).

**Fig. 5:**
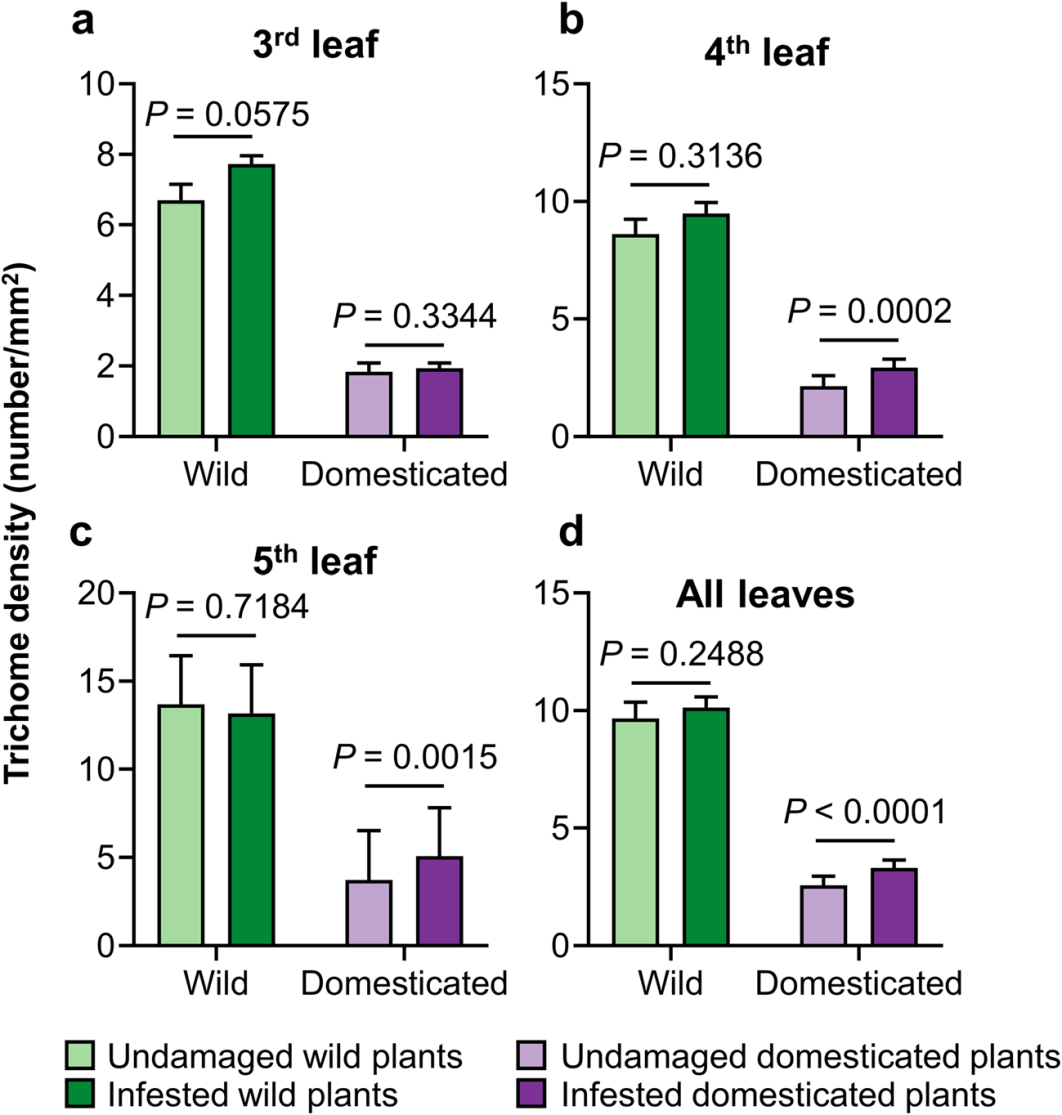
Feeding by *Diabrotica balteata* adults induced higher trichome density in domesticated *Cucurbita argyrosperma* plants. Trichome density of squash plants was recorded ten days after four-day period of infestation (four hours per day) by two *D. balteata* adults. Control plants were kept uninfested. Trichome density on the adaxial side of the newly emerged 3rd, 4th, and 5th leaves (**a**, **b**, and **c**) of wild populations and domesticated varieties was presented as the number of trichomes per square millimeter leaf area. Error bars represent standard error of the mean (undamaged wild plants, *n* = 3 population × 3 replicates; infested wild plants, *n* = 3 population × 5 replicates; undamaged domesticated plants, *n* = 3 varieties × 3 replicates; infested domesticated plants, *n* = 3 varieties × 5 replicates). *P* values are given for comparisons among treatments using mixed generalized linear model with Gamma distribution and log link function followed by Tukey post-hoc test

When data were pooled within wild and domesticated plants, domestication status accounted for the variance in trichome induction (Chisq = 4.9364, df = 1, *P* = 0.02630). Foliar feeding by adult *D. balteata* beetles did not lead to an increase in trichome density in the third leaf for domesticated varieties (Tukey test: *P* = 0.3344), but there is a small trend of induction in wild populations after the beetles feeding (Tukey test: *P* = 0.0575) (Fig. 5a). In contrast, herbivory did not increase trichome density in the fourth and fifth leaves in wild plants (undamaged—infested in 4th leaf, *P* = 0.3136; undamaged—infested in 5th leaf, *P* = 0.7184), but significantly induced trichome accumulation in domesticated plants (undamaged—infested in 4th leaf, *P* = 0.0002; undamaged—infested in 5th leaf, *P* = 0.0015) (Fig. 5b-c). Foliar feeding did not result in increased trichome density for wild populations (*P* = 0.2488) but significantly induced trichome accumulation for domesticated plants (*P* < 0.0001) (Fig. 5d).

## Discussion

In this study, we first analyzed the chemical and physical defensive traits in different tissues of wild and domesticated plants of *C. argyrosperma*. Subsequently, we investigated whether belowground and aboveground herbivory induced these defenses in the roots and leaves of *C. argyrosperma* with different domestication statuses. Our findings revealed that domesticated *C. argyrosperma* had lower levels of cucurbitacin in roots and cotyledons compared to their wild counterparts, along with reduced trichome density on leaves. We also found activation of cucurbitacin biosynthesis genes by root feeding of *D. balteata* larvae and the trichome formation in response to foliar feeding by its adults. These inductions of defensive responses varied among varieties, yet domestication significantly enhanced trichome induction in response to herbivory. These findings support one of our hypotheses, suggesting that the enhancement of inducible defenses is only evident in domesticated plants compared to their wild counterparts.

In a previous study, we found a complete loss of cucurbitacin biosynthesis in both roots and cotyledons of two *C. argyrosperma* varieties domesticated for ornamental use (Jaccard et al. 2023). Here, we corroborated these findings in three additional domesticated varieties (OCS, OCT, and OCW), demonstrating that domestication leads to cucurbitacin depletion in the roots and cotyledons of *C. argyrosperma* (Fig. 1). Indeed, the reduction or loss of cucurbitacin biosynthesis during domestication has been observed in many Cucurbitaceae plants (Shang et al. 2014; Zhou et al. 2016; Pickersgill 2018; Brzozowski et al. 2020). The loss of cucurbitacin biosynthesis in domesticated squash may be attributed to impaired expression of genes associated with cucurbitacin biosynthesis (Jaccard et al. 2023). Consistent with our previous findings, the transcription levels of cucurbitacin biosynthetic genes in domesticated varieties was notably lower than that in wild squash populations (Figs. S3 and 3).

Some recent studies suggest that cucurbitacins are constitutive defensive compounds that cannot be induced by herbivory (Milano et al. 2015; Brzozowski et al. 2020; Bruno et al. 2023). However, there are also contrary examples that demonstrate the inducibility of cucurbitacin in response to herbivory (Tallamy 1985; Agrawal et al. 1999). In this study, belowground infestation by *D. balteata* larvae failed to increase cucurbitacin contents in roots of both wild populations and domesticated varieties of squash (Fig. 2). Interestingly, the transcription of several critical cucurbitacin biosynthetic genes in some squash cultivars was significantly induced by root herbivory (Fig. 3), indicating that the process of cucurbitacin biosynthesis was activated. It is plausible that cucurbitacin biosynthesis is time-dependent and occurs late during root herbivory, which could explain why the increase in cucurbitacin content was not detected in our 24-h infestation experiment. An alternative explanation could be that the tradeoff between growth and defense accelerates cucurbitacin degradation, thereby hindering further accumulation of cucurbitacin under root herbivory. The induction pattern of key genes (*Carg11552*, *Carg06672*, and *Carg07313*) involved in cucurbitacin biosynthesis showed a similar pattern between wild and domesticated squash (Fig. 3), suggesting that the sensitivity of these genes to root herbivory is independent of domestication status.

Trichome density was lower in domesticated plants than in wild plants (Fig. 4a-b). Since no cucurbitacins were detected in the leaves of both wild and domesticated plants, we asked whether trichomes could act as a barrier to deter leaf-feeding *Diabrotica balteata* beetles. We expected that wild squash populations with higher trichome density would suffer less leaf damage than plants of domesticated varieties. However, although leaf consumption by *D. balteata* beetles was different among plants, there was a trend of higher leaf damage on wild populations compared to domesticated varieties (Fig. 4c-d). Since we only identified simple and non-glandular trichomes on squash leaves, it appears that the physical protection provided by these trichomes does not significantly influence the feeding of *D. balteata* beetles. Trichome-based plant defenses are highly specific to both the plant and the herbivores (Hare 2005; Tian et al. 2012), and high trichome density may not necessarily correlate with plant resistance to well-adapted herbivores (Chen et al. 2020). For example, the striped cucumber beetle *Acalymma vittatum* did not show any preference between bottle gourd *Lagenaria siceraria* and cucumber *Cucumis sativa* when the two plant species with different amounts and densities of foliar trichomes were grown together in common garden experiments (Kaur and Kariyat 2023). Our previous study also showed that the performance of *Spodoptera latifascia* caterpillars does not vary among domesticated varieties of squash with differing densities of trichomes (Jaccard et al. 2021). Given that both *D. balteata* and squash originate from Mesoamerica, it is possible that *D. balteata* beetles have evolved to overcome the trichome-based defense of squash plants during their long history of co-evolution.

Many plants increase their trichome formation in new leaves in response to foliar damage (Agrawal 1999; Brian Traw and Dawson 2002; Dalin and Björkman 2003; Tian et al. 2012). In a particular study, the induction of trichome formation was found to correlate with the domestication status of plants. For instance, a cultivated tomato (var. Better Boy) has been found to present higher inducibility of leaf trichomes following herbivore damage compared to wild tomato *Solanum pimpinellifolium* L. (accession LA 2093) and cherry tomato *S. lycopersicum* L. var. *cerasiforme* (accession Matts Wild Cherry) (Paudel et al. 2019). Here, we found that leaf feeding by *D. balteata* beetles induced the formation of trichomes on fresh leaves of one wild population (Walt) and two domesticated varieties (OCS and OCW) (Fig. S4), suggesting the inducibility of this physical trait in squash is cultivar-specific. Domesticated plants significantly increased trichome density in response to herbivory, especially in younger leaves (Fig. 5). These results might be explained by the genetic changes in cultivated plants resulting from genetic drift during domestication (Chen et al. 2015). Genetic changes likely influence the regulation of genes associated with trichome formation, enhancing their expression in response to herbivory. The reduction of constitutive trichome density and the increased investment in inducible trichome formation in domesticated plants may also be driven by the trade-off between growth and defense. For example, selection for increased fruit size during domestication leads to a lower concentration and diversity of phenolics in apple, indicating an allocation trade-off between growth and defense that plays a role during domestication (Whitehead and Poveda 2019). However, domestication does not lead to a robust allocation trade-off in crops (Turcotte et al. 2014). In cereals, domestication reduces silicon-based defenses, but the size-standardized growth rate is independent of domestication status and does not exhibit a trade-off with silicon or phenolic defense (Simpson et al. 2017). More wild populations and domesticated varieties of squash should be tested in future experiments to verify the correlation between domestication status and inducible trichome formation.

In conclusion, our results indicated that domestication reduces the constitutive chemical and physical defenses in both roots and leaves of squash. The inducible defenses of squash in response to belowground and aboveground infestation were cultivar-specific, but unlike cucurbitacin biosynthetic gene induction, the trichome induction was more affected by domestication status.

## Supplementary Information

Below is the link to the electronic supplementary material.

Supplementary file1 (DOCX 13346 KB)

Fig. S4 (PNG 364 kb)

High resoluton image (TIF 779 KB)

## Data Availability

No datasets were generated or analysed during the current study.
