# Supplementary material for: Cultivar-Specific Defense Responses in Wild and Cultivated Squash Induced by Belowground and Aboveground Herbivory
Source: J Chem Ecol. 2024 Jun 24;50(11):738–50. doi: 10.1007/s10886-024-01523-9 (PMC11543723; doi:10.1007/s10886-024-01523-9)
Supplement: Supplementary file 1 — Supplementary file1 (DOCX 13346 KB) [file 10886_2024_1523_MOESM1_ESM.docx]

**Supplementary Information**

**
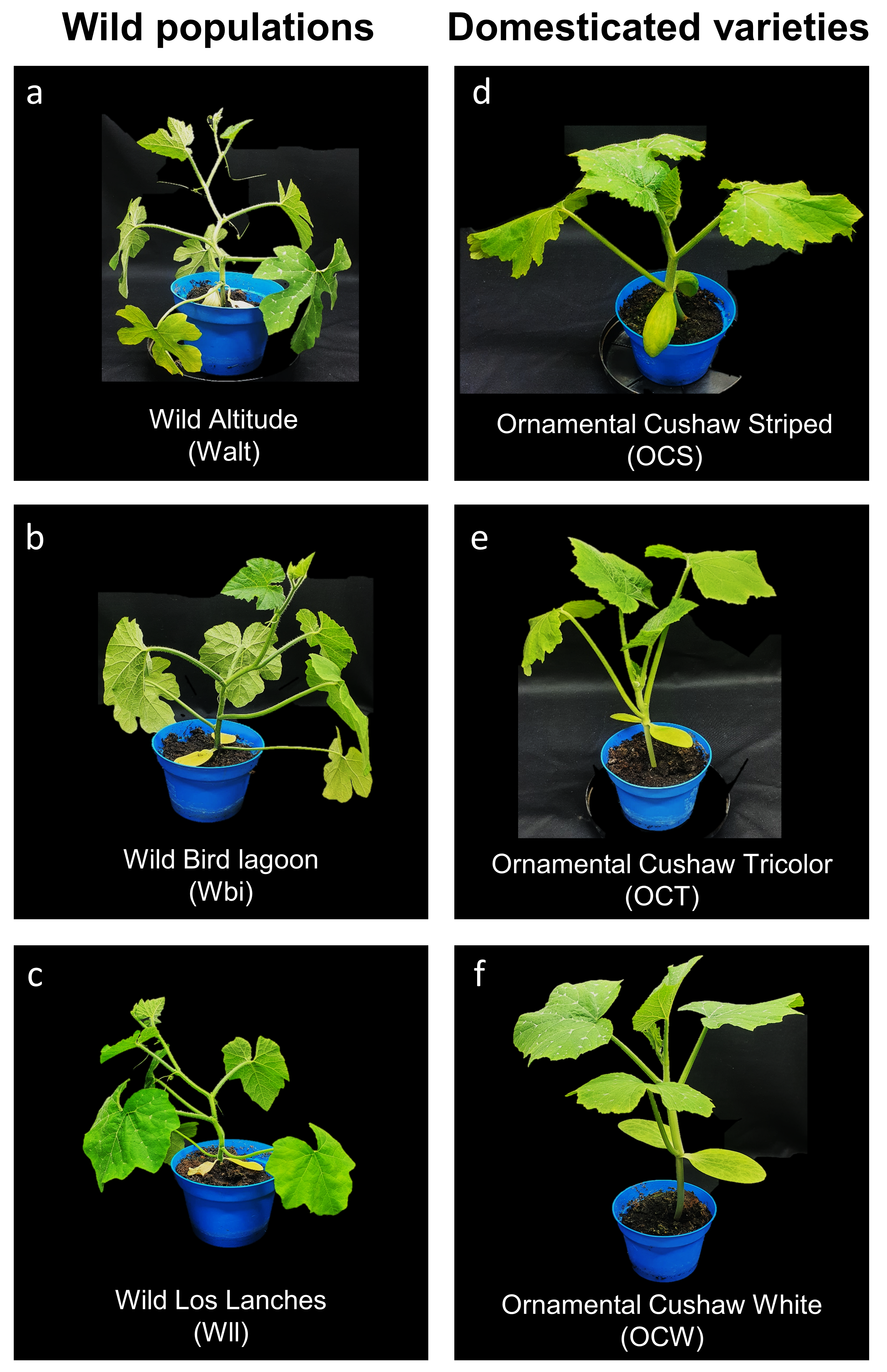
**

**Figure S1** Three-week old plants of wild populations (**a**) Wild Altitude (Walt), (**b**) Wild Bird lagoon (Wbi), and (**c**) Wild Los Lanches (Wll) and ornamental varieties (**d**) Ornamental Cushaw Orange Striped (OCS), (**e**) Ornamental Cushaw Tricolor (OCT), and (**f**) Ornamental Cushaw White (OCW).

**
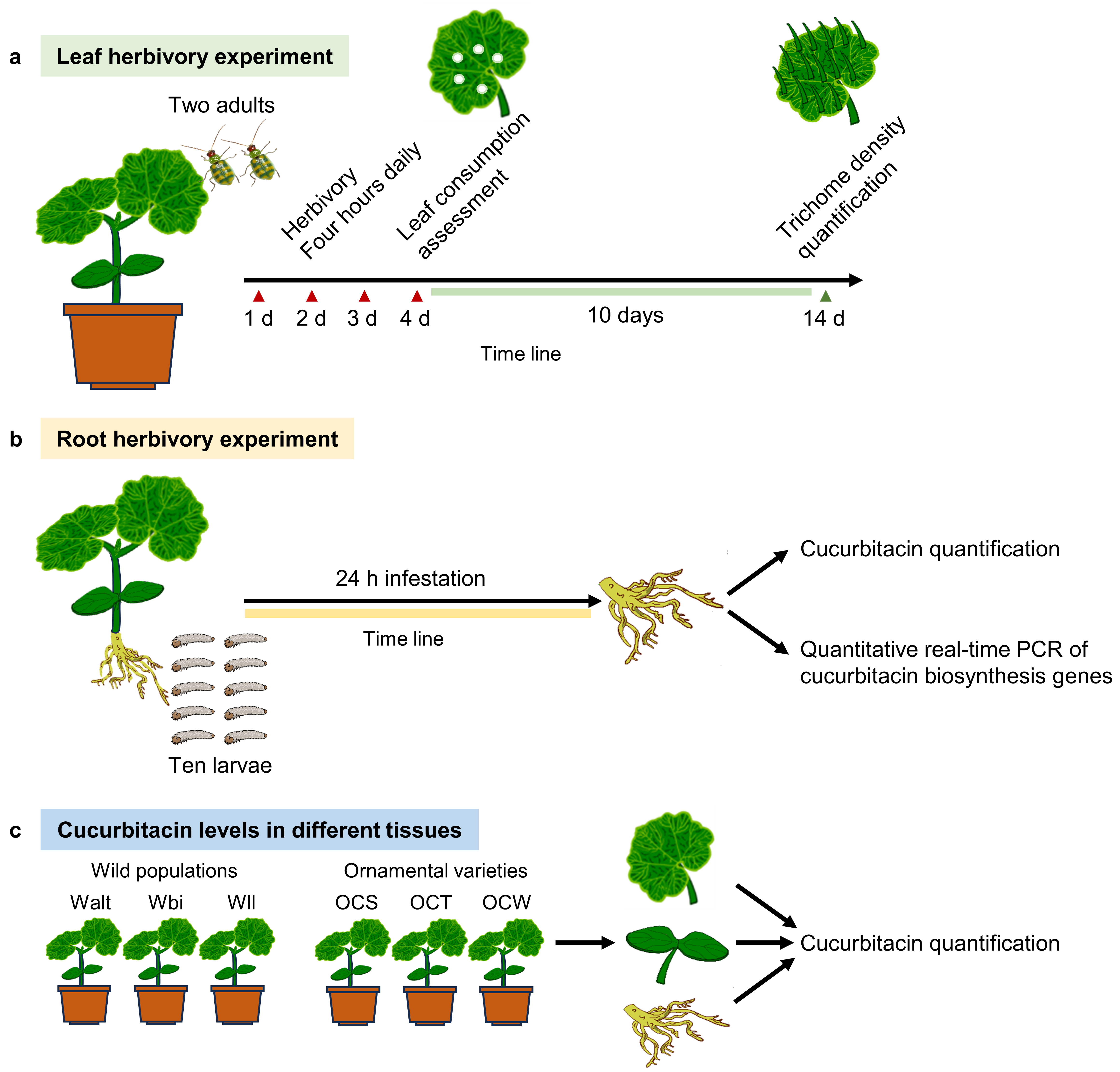
**

**Figure S2** Illustration of the two methods used to treat squash plants with leaf and root feeders. Leaf herbivory experiment: (**A**) Two adults of *Diabrotica balteata,* one week post-emergence from pupae, were allowed to feed on leaves and cotyledons for four hours daily. After four days, photographs of the two exposed leaves were taken, and leaf consumption by *D. balteata* adults was assessed using ImageJ. Ten days after the four-day herbivory period, the trichome density of the three new leaves of each plant was determined. Root herbivory experiment: (**B**) Ten second-instar larvae were released onto the soil surface around the stem of each plant to infest the roots. After 24 h infestation, the entire root system was harvested for cucurbitacin quantification and quantitative real-time PCR of cucurbitacin biosynthesis genes. Control plants remained uninfested. (**C**) Leaves, cotyledons and roots of three wild populations (Walt, Wbi, and Wll) and three ornamental varieties (OCS, OCT, and OCW) were used to quantify cucurbitacin levels in different tissues of uninfested plants. Fifteen-day-old squash plants with two expanded leaves were used in these experiments.

**
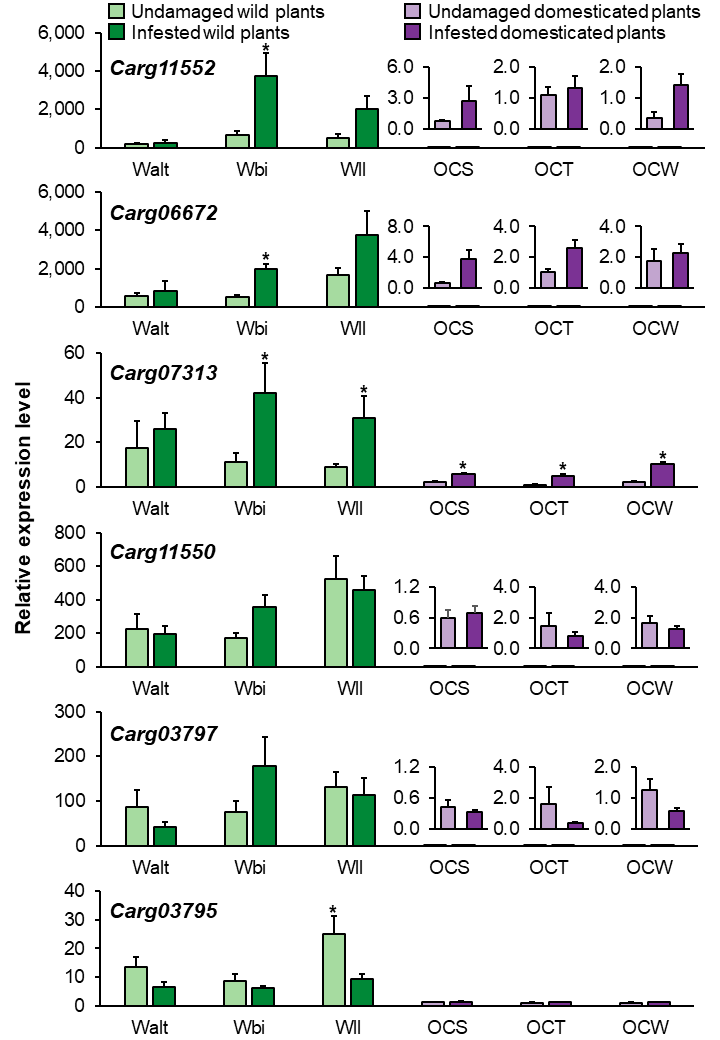
**

**Figure S2** Relative gene expression of six cucurbitacin biosynthesis genes in roots of wild and domesticated squash plants. Mean transcript levels (+SE) of cucurbitacin biosynthesis genes in roots of three wild populations (Walt, Wbi, and Wll) and three domesticated varieties (OCS, OCT, and OCW) of squash plants that were not infested (undamaged control, *n* = 3) or infested with 10 second-instar *Diabrotica balteata* larvae for 24 h (infested plants, *n* = 5). Asterisks indicate significant differences between control and root infestation. (* *p* < 0.05, Student’s *t*-test).


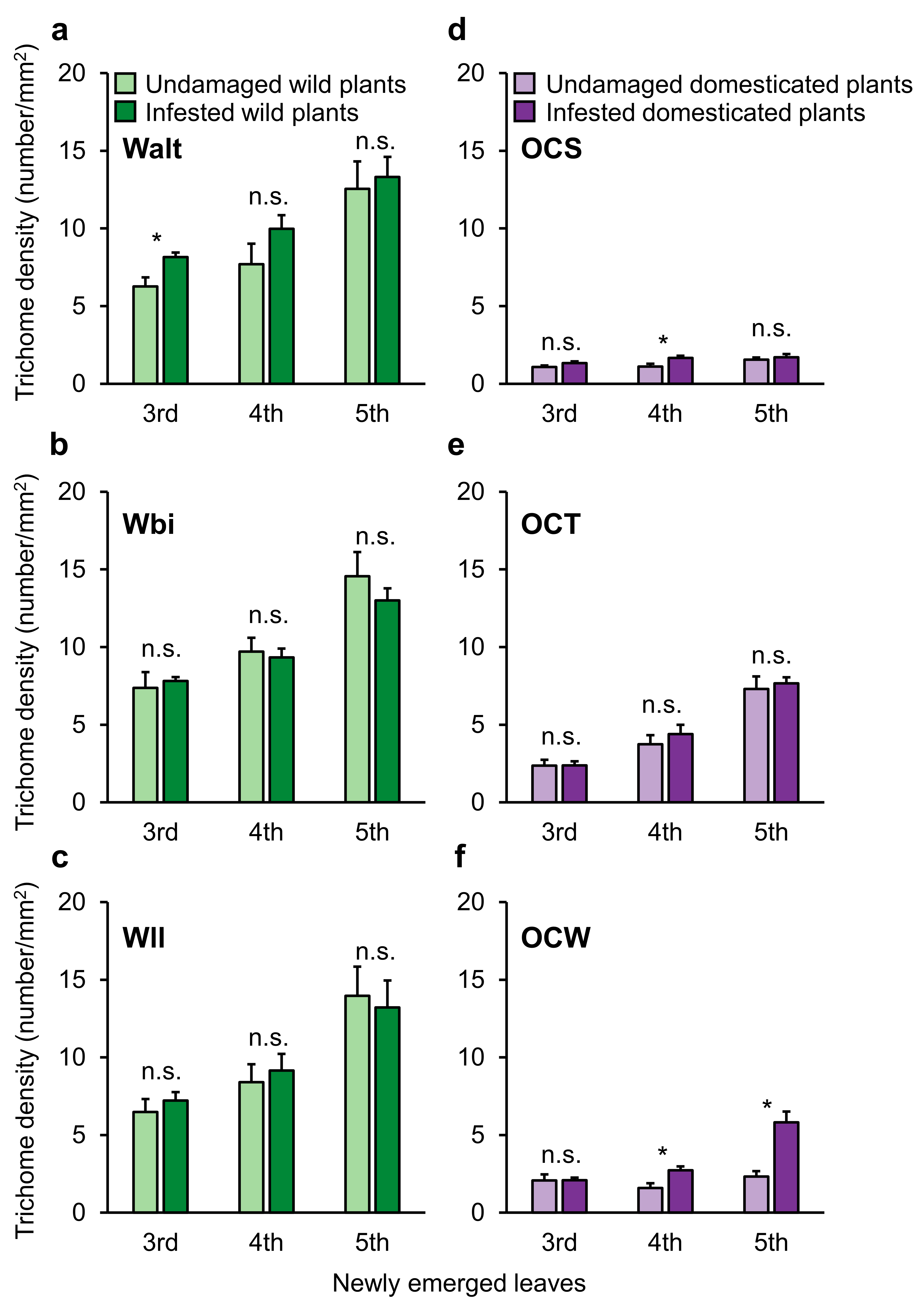


**Figure S3** Effects of feeding by *Diabrotica balteata* adults on trichome density in *Cucurbita argyrosperma* plants. Trichome density of squash plants was determined ten days after a four-day period of infestation (four hours per day) with two *D. balteata* adults. Control plants remained uninfested. Trichome density on the adaxial side of the newly emerged leaves (3^rd^, 4^th^, and 5^th^ leaves) of three wild populations Walt (**a**), Wbi (**b**), Wll (**c**) and three ornamental varieties OCS (**d**), OCT (**e**), OCW (**f**) are presented as the number of trichomes per square millimeter leaf area. Error bars represent standard error of the mean (for controls, *n* = 3; for leaf infestation, *n* = 5). Significant differences in trichome density per leaf between control and infested plants for each variety are indicated by asterisks (* *p* < 0.05, Student’s *t*-test). Not significant (N.S., *p* > 0.05).

| **Table S1** Genes involved in the cucurbitacin biosynthesis pathway in *Cucumis sativus*, *Cucumis melo*, and *Citrullus lanatus* and orthologues found in  *Cucurbita argyrosperma*. | | | | |
| --- | --- | --- | --- | --- |
| **Query Gene in cucumber (*Cucumis sativus*)** | **Gene name** | **Orthologs in squash (*Cucurbita argyrosperma*)** | **BlastP Identity (%)** | **Annotation (function in cucumber)** |
| ***Csa6G088160*** | *CYP81Q58* | *Carg03795* | 78.15 | Cytochrome P450 enzyme; C25 hydroxylase |
| ***Csa6G088170*** | *CYP89A140* | *Carg11550* | 76.48 | Cytochrome P450 enzyme |
| ***Csa6G088690*** | *Bi* | *Carg11552* | 87.13 | Cucurbitadienol synthase; oxidosqualene cyclase gene family |
| ***Csa6G088700*** | *ACT* | *Carg03796* | 68.76 | Acetyltransferase |
| ***Csa6G088710*** | *CYP87D19* | *Carg03797* | 83.82 | Cytochrome P450 enzyme |
| ***Csa1G044890*** | *CYP87D20* | *Carg06672* | 89.24 | Cytochrome P450 enzyme; C11 hydroxylase + C20 hydroxylase |
| ***Csa3G698490*** | *CYP712D8* | *Carg07313* | 86.19 | Cytochrome P450 enzyme |
| ***Csa3G903540*** | *CYP88L2* | *Carg14872* | 38.28 (low identity) | Cytochrome P450 enzyme; C19 hydroxylase |
| ***Csa3G903550*** | *CYP88L3* | *Carg08824* | 41.94 (low identity) | Cytochrome P450 enzyme |
| *Csa5G156220* | *Bl* | *Carg16672* | 61.58 | leaf-specific helix-loop-helix (bHLH) transcription factor |
| *Csa5G157230* | *Bt* | *Carg02488* | 61.54 | Fruit-specific helix-loop-helix (bHLH) transcription factor |
| **Query Gene in melon (*Cucumis melo*)** | **Gene name** | **Orthologs in squash (*Cucurbita argyrosperma*)** | **BlastP Identity (%)** | **Annotation (function in melon)** |
| ***Melo3C022377*** | *Cm160* | *Carg03795* | 80.71 | Cytochrome P450 enzyme |
| ***Melo3C022376*** | *Cm170* | *Carg11550* | 77.97 | Cytochrome P450 enzyme |
| ***Melo3C022375*** | *Cm180* | *Carg11551* | 77.08 | Cytochrome P450 enzyme; C2 hydroxylase |
| ***Melo3C022374*** | *CmBi* | *Carg11552* | 88.6 | Cucurbitadienol synthase; oxidosqualene cyclase gene family |
| ***Melo3C022373*** | *CmACT* | *Carg03796* | 71.01 | Acetyltransferase |
| ***Melo3C022372*** | *Cm710* | *Carg03797* | 84.63 | Cytochrome P450 enzyme |
| ***Melo3C002192*** | *Cm890* | *Carg06672* | 89.45 | Cytochrome P450 enzyme; C11 hydroxylase + C20 hydroxylase |
| ***Melo3C023960*** | *Cm490* | *Carg07313* | 87.22 | Cytochrome P450 enzyme |
| *Melo3C005611* | *CmBt* | *Carg16672* | 60.83 | Fruit-specific helix-loop-helix (bHLH) transcription factor |
| *Melo3C005610* | *CmBr* | *Carg02488* | 67.43 | Root-specific helix-loop-helix (bHLH) transcription factor |
| **Query Gene in watermelon (*Citrullus lanatus*)** | **Gene name** | **Orthologs in squash (*Cucurbita argyrosperma*)** | **BlastP Identity (%)** | **Annotation (function in watermelon)** |
| ***Cla007077*** | *Cl160* | *Carg03795* | 85.31 | Cytochrome P450 enzyme |
| ***Cla007078*** | *Cl170* | *Carg11550* | 78.94 | Cytochrome P450 enzyme |
| ***Cla007079*** | *Cl180* | *Carg11551* | 80.34 | Cytochrome P450 enzyme; C2 hydroxylase |
| ***Cla007080*** | *ClBi* | *Carg11552* | 92.02 | Cucurbitadienol synthase; oxidosqualene cyclase gene family |
| ***Cla007081*** | *ClACT* | *Carg03796* | 74.94 | Acetyltransferase |
| ***Cla007082*** | *Cl710* | *Carg03797* | 89.42 | Cytochrome P450 enzyme |
| ***Cla008355*** | *Cl890A* | *Carg06672* | 82.49 | Cytochrome P450 enzyme; C11 hydroxylase + C20 hydroxylase |
| ***Cla008354*** | *Cl890B* | *Carg06672* | 90.08 | Cytochrome P450 enzyme; C11 hydroxylase + C20 hydroxylase |
| ***Cla017252*** | *Cl490* | *Carg07313* | 88.68 | Cytochrome P450 enzyme |
| ***Cla016164*** | *Cl510* | *Carg08824* | 92.6 | Cytochrome P450 enzyme |
| *Cla011508* | *ClBt* | *Carg16672* | 64.84 | Fruit-specific helix-loop-helix (bHLH) transcription factor |
| *Cla011510* | *ClBr* | *Carg02488* | 62.35 | Root-specific helix-loop-helix (bHLH) transcription factor |

| **Table S2** Primers used for real-time qPCR. | |  |
| --- | --- | --- |
| Gene ID in CuGenDB | Primer (Forward) | Primer (Reverse) |
| *Carg11552* | AGATGGAGGGTGGGGTCTAC | TCTCCAAGCAGCCTTAGTGC |
| *Carg03795* | GCCCACTTCTACTGCCACAT | CATCCCCACCCTCAAACCTC |
| *Carg11550* | CCCTTCTCGCAAATGGGTCT | CTAAACATGGCGTGGCGAAG |
| *Carg03797* | AGCCGTCCGTCTGTTGAAAT | TTCAACGGGACCGCCATAAA |
| *Carg07313* | CGAAATTCGCGTTCGGAGAC | TGATCGCTCAACTTGTCGCT |
| *Carg07314* | CGCGCATTATCGCAAACAGA | AGGAGTGTCTCCGGCTGTAT |
| *Carg06672* | CGCTGGCGGTTGCTTATTAC | AGCCAACTGAAGCGTCTCTC |

**Table S3** Putative identification of cucurbitacins found in roots and cotyledons of wild and domesticated squash. Cucurbitacins were putatively identified based on their exact masses (allowing for molecular formula determination) and retention times and compared with those of the standard Cucurbitacin B as well as with available databases such as the Dictionary of Natural Product (CRC Press).

| **RT (min)** | **(M+HCOO)^-^** | **(M-H)^-^** | **MF** | **PI** |
| --- | --- | --- | --- | --- |
| 3.79 | 603.3174 | 557.2624 | C32H46O8 | Cucurbitacin B |
| 2.52 | 723.3651 | 677.3601 | C36H54O12 | Unknown Cucurbitacin |
| 2.78 | 765.3694 | 719.364 | C38H56O13 | Cucurbitacin B glucoside (or isomer) |
| 3.02 | 765.3694 | 719.364 | C38H56O13 | Cucurbitacin B glucoside (or isomer) |
| 3.08 | 767.3834 | 721.3779 | C38H58O13 | Dihydrocucurbitacin B glucoside (or isomer) |

RT: retention time; MF: molecular formula; PI: putative identification.
