# Supplementary figures and images for: Cultivar-Specific Defense Responses in Wild and Cultivated Squash Induced by Belowground and Aboveground Herbivory
Source: J Chem Ecol. 2024 Jun 24;50(11):738–50. doi: 10.1007/s10886-024-01523-9 (PMC11543723; doi:10.1007/s10886-024-01523-9)

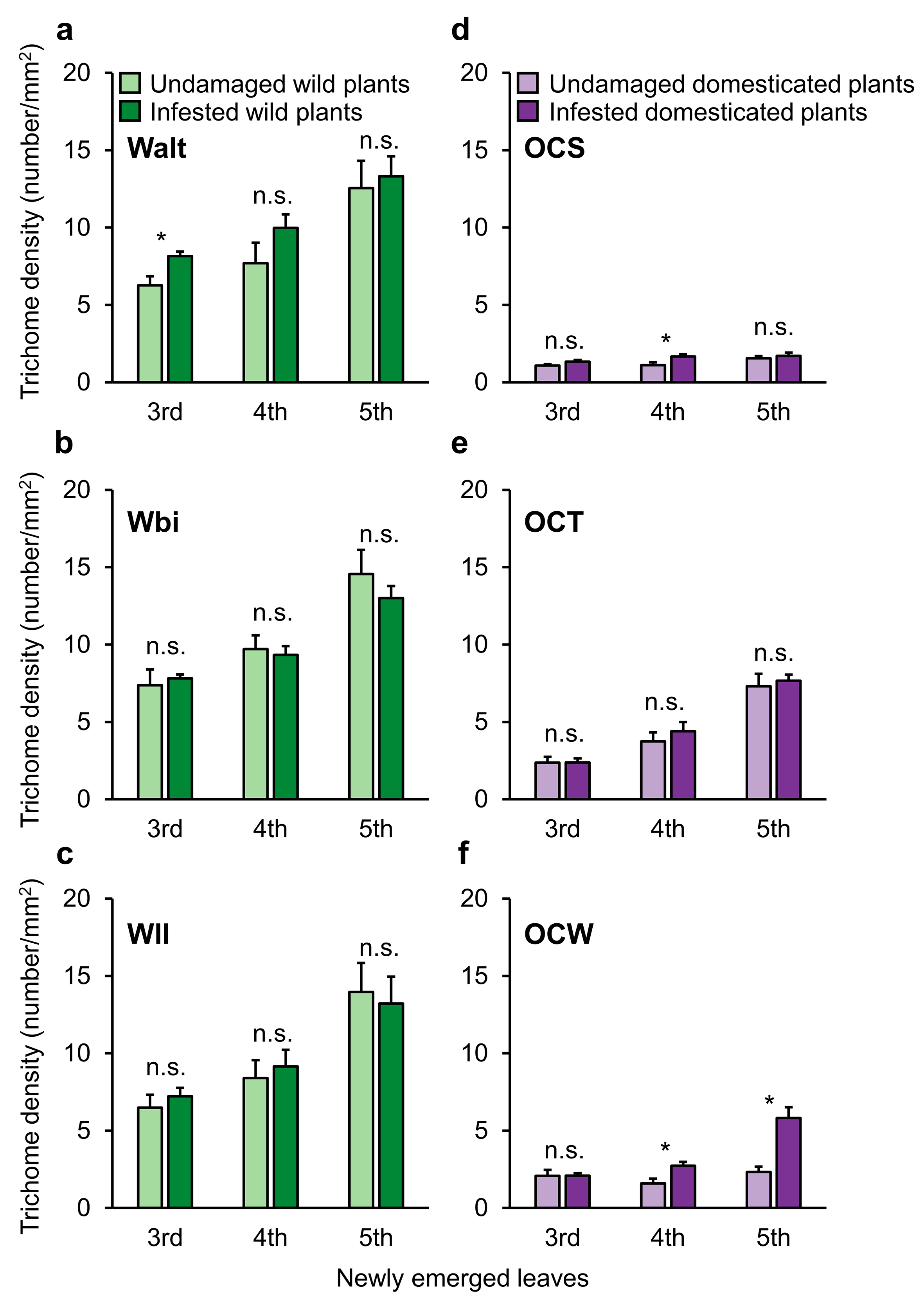

Supplement: Supplementary file 2 — Fig. S4 (PNG 364 kb) [file 10886_2024_1523_Fig6_ESM.png]

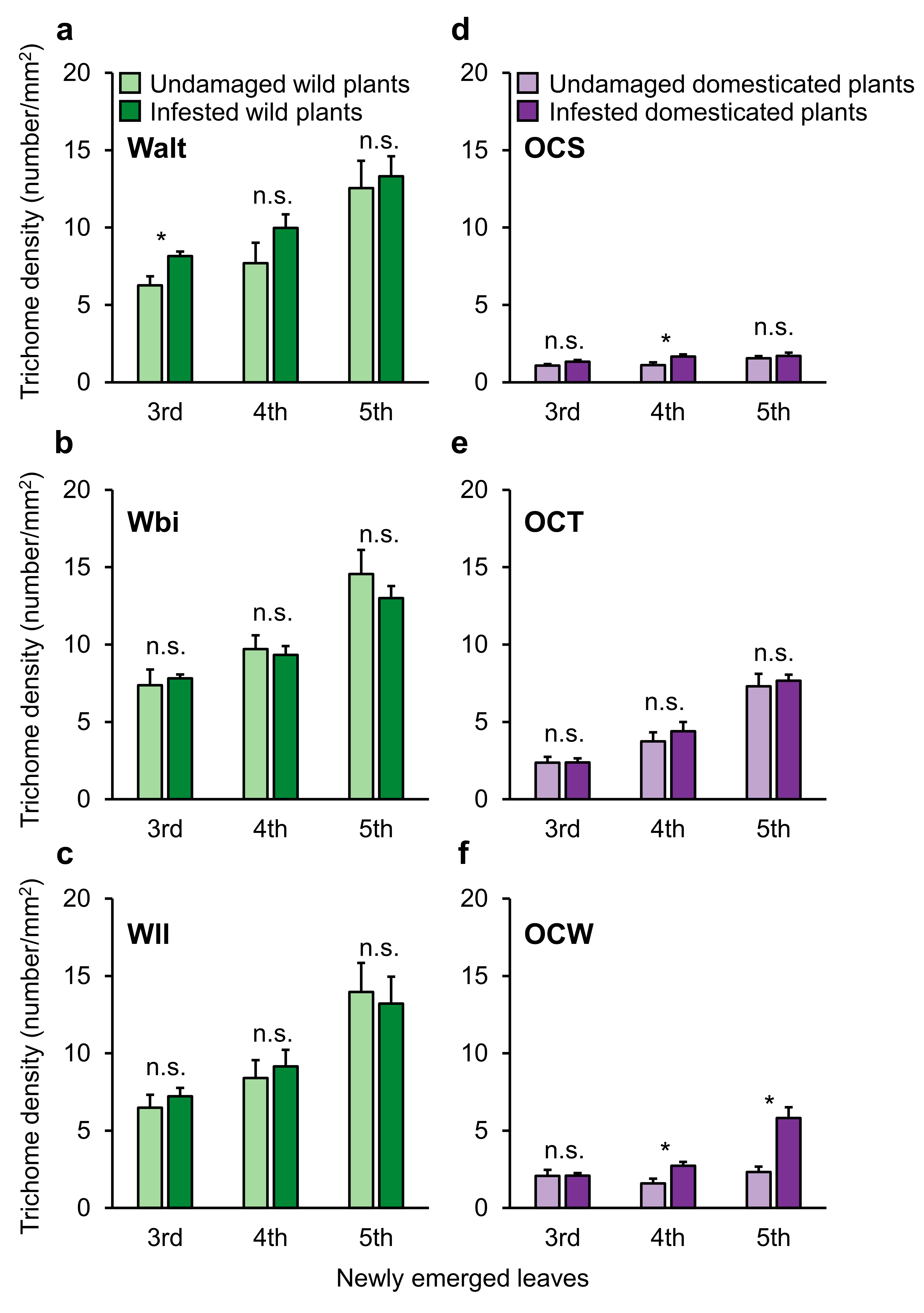

Supplement: Supplementary file 3 — High resoluton image (TIF 779 KB) [file 10886_2024_1523_MOESM2_ESM.tif]
